# Diagnosis of pancreaticobiliary malignancy by detection of minichromosome maintenance protein 5 in biliary brush cytology

**DOI:** 10.1038/bjc.2016.447

**Published:** 2017-01-12

**Authors:** Margaret G Keane, Matthew T Huggett, Michael H Chapman, Gavin J Johnson, George J Webster, Douglas Thorburn, James Mackay, Stephen P Pereira

**Affiliations:** 1 Institute for Liver and Digestive Health, University College London, Royal Free Campus, Pond St, London NW3 2PF, UK; 2 Department of Gastroenterology, University College London Hospitals NHS Foundation Trust, 235 Euston Road, London NW1 2BG, UK; 3 Department of Genetics, Evolution and Environment, University College London, Gower St, London WC1E 6BT, UK

**Keywords:** pancreatic ductal adenocarcinoma, cholangiocarcinoma, endoscopic retrograde cholangiopancreaticography, chronic pancreatitis, primary sclerosing cholangitis, IGG4-related disease, minichromosome maintenance replication proteins

## Abstract

**Background:**

Biliary brush cytology is the standard method of evaluating biliary strictures, but is insensitive at detecting malignancy. In pancreaticobiliary cancer minichromosome maintenance replication proteins (MCM 2–7) are dysregulated in the biliary epithelium and MCM5 levels are elevated in bile samples. This study aimed to validate an immunocolorimetric ELISA assay for MCM5 as a pancreaticobiliary cancer biomarker in biliary brush samples.

**Methods:**

Biliary brush specimens were collected prospectively at ERCP from patients with a biliary stricture. Collected samples were frozen at −80 °C. The supernatant was washed and lysed cells incubated with HRP-labelled anti-MCM5 mouse monoclonal antibody. Test positivity was determined by optical density absorbance. Patients underwent biliary brush cytology or additional investigations as per clinical routine.

**Results:**

Ninety-seven patients were included in the study; 50 had malignant strictures. Median age was 65 years (range 21–94) and 51 were male. Compared with final diagnosis the MCM5 assay had a sensitivity for malignancy of 65.4% compared with 25.0% for cytology. In the 72 patients with paired MCM5 assay and biliary brush cytology, MCM5 demonstrated an improved sensitivity (55.6% *vs* 25.0% *P*=0.0002) for the detection of malignancy.

**Conclusions:**

Minichromosome maintenance replication protein5 is a more sensitive indicator of pancreaticobiliary malignancy than standard biliary brush cytology.

Being able to diagnose pancreatic and biliary tract cancer with a single investigation and at an early stage when curative treatment is feasible remains a significant clinical challenge. Current diagnostic modalities such as serum tumour markers, cross-sectional imaging and cholangiography do not have sufficient sensitivity to diagnose malignancy when used alone or in combination, especially in the early stages of malignancy when the lesion is small (Lee, 2006; Kalaitzakis *et al*, 2011). Cytological or histological confirmation is almost always required prior to surgical resection or treatment and are usually acquired from biliary strictures or associated masses, commonly by brush cytology or endobiliary biopsy (De Bellis *et al*, 2002). Although these techniques do not compromise resection margins in potentially resectable cases and have a high specificity (96–100%) for malignancy, sensitivity remains low (9–57%) and patients frequently have to undergo multiple procedures to obtain a final diagnosis (De Bellis *et al*, 2002; Harewood *et al*, 2004; Moreno Luna *et al*, 2006).

The poor sensitivity of cytology is multifactorial, biliary tract and pancreatic cancers typically have a desmoplastic component and specimens are often paucicellular in nature. In addition obtaining and interpreting cytological specimens can be operator- and observer-dependent. Therefore there has been a growing interest in developing adjunctive tests such as fluorescence *in situ* hybridisation (FISH) and evaluating novel procedures, such as cholangioscopy and confocal endomicroscopy, to improve the detection of pancreaticobiliary malignancy (Kalaitzakis *et al*, 2012; Keane *et al*, 2013). A recent meta-analysis found the sensitivity of endoscopic ultrasound-guided fine needle aspiration (EUS-FNA) to be 74.2%, FISH 54.2%, serum CA19-9 69.3% and a K-ras mutation 47.0% (Burnett *et al*, 2014). Randomised controlled trials are ongoing to determine if multiple endobiliary brushings or multimodal stricture assessment including cholangioscopy and FISH are the optimal approach for the assessment of indeterminate biliary strictures (Cote *et al*, 2015).

The initiation of DNA replication is the final part of growth regulation, located downstream of all growth regulatory pathways (Williams and Stoeber, 1999). Minichromosome maintenance proteins (MCM2–7) participate in the assembly of prereplicative complexes, which initiate DNA synthesis. All six MCM proteins are essential for replication and are present in all phases of the proliferative cell cycle but are tightly downregulated in the quiescent, terminally differentiated ‘out-of-cycle' states. The presence of one MCM protein reflects the presence of the other five as all six are loaded together onto DNA as a heterohexamer on exit from metaphase (Blow and Hodgson, 2002). We have shown that these biomarkers detect, in addition to actively proliferating malignant cells, those with growth potential, that is, premalignant cells, as dysregulation in MCM proteins is an early event in epithelial carcinogenesis (Freeman *et al*, 1999; Stoeber *et al*, 2001). Moreover, we have demonstrated the utility of these novel biomarkers of growth as diagnostic markers in a range of cancers including biliary tract and pancreatic cancer (Wharton *et al*, 2001; Williams and Stoeber, 2007; Ayaru *et al*, 2008; Loddo *et al*, 2009).

## Study aim

The aim of the study was to validate a newly developed immunocolorimetric ELISA test for the MCM5 protein in samples obtained by biliary brush cytology.

## Materials and methods

### Setting

A large regional Hepatopancreaticobiliary (HPB) centre. Endoscopic procedures were performed at University College London Hospital (UCLH) or the Royal Free Hospital (RFH), London.

### Design

The design was a Prospective Cohort Study.

### Ethical approval

The study was approved by the Joint UCLH/UCL ethical committee and all patients gave written informed consent (NRES: 06/Q0512/106).

### Inclusion criteria

Patients >18 who underwent a clinically indicated endoscopic retrograde cholangiopancreatography (ERCP) between 01 June 2011 and 30 June 2015.

### Clinical data

For each patient recruited, the electronic medical records were reviewed and information was recorded in an electronic spreadsheet. Data were recorded from the Pathology (CoPath histology database, Sunquest, Tucson, AZ, USA), Endoscopy (GI reporting tool, Unisoft Medical Systems, Enfield, UK) and Imaging (PACS: picture archiving and communication system, GE Healthcare, Pollards Wood, UK) database systems. Data collected included demographic information (age, sex and hospital number), history of acute or chronic pancreatitis or malignancy, family history of pancreatic cancer or relevant clinical syndrome. Cross-sectional imaging (computed tomography (CT) and/or magnetic resonance cholangiopancreatography (MRCP)) features were recorded. Details of the ERCP procedure along with cytology and histology results were recorded. For patients referred for surgery, date of the operation, type of resection and final histology were recorded. Length of follow-up was calculated from first procedure to last clinic appointment attended, or date of clinic discharge, or death.

A diagnosis of malignancy was made by surgical resection, positive histological biopsy or positive cytology where available, or evidence of disease progression on imaging when not. Benign disease was established by negative pathology and a median of 14 (range 0–49) months clinical follow-up.

### Patients

Between June 2011 and June 2015, 102 patients with an established or indeterminate biliary stricture were invited to participate in the study. Established patients were defined as those with an existing diagnosis for their biliary stricture who were having a repeat ERCP for therapeutic reasons such as stent change and consented to participate in the study. Patients with an indeterminate biliary stricture included patients with a stricture on cross-sectional imaging; 41% (28 out of 68) had at least one ERCP with biliary brushing prior to entering the study (11 had one prior ERCP, 14 two prior ERCPs, 3 three prior ERCPs and in one case four prior ERCPs before entering the study).

Five patients were excluded as there was insufficient sample remaining to perform the MCM5 test following prior investigations. Ninety-seven patients were included in the final analysis.

### Procedures

#### Endoscopic retrograde cholangiopancreatography

The procedures were performed under general anaesthesia or conscious sedation with midazolam and fentanyl. All ERCPs were performed by one of five experienced endoscopists using a standard therapeutic duodenoscope (JF; Olympus, Southend-on-Sea, UK). All procedures were performed in the endoscopy unit with fluoroscopy. Biliary brush cytology was obtained following cholangiography using a wire-guided sheathed cytology brush (Combocath, Microinvasive; Boston Scientific, Notick, MA, USA), advanced across the stricture several times before being resheathed and the sheathed brush withdrawn from the endoscope. In this study, tissue acquisition was performed before any therapy (stricture dilation, stenting and so on.) In 72 cases, where clinically indicated, a single biliary brush sample was taken for routine cytology before another biliary brush sample was taken for the MCM5 assay.

The clinical cytology specimen was then transferred immediately to glass slides by smearing the cellular material from the brush directly onto two slides. These were fixed and later stained for malignant cells using the standard Papanicolaou technique. Brush cytology samples were analysed by expert cytopathologists within the context of a multidisciplinary cancer review meeting. Cytology was classified as malignant or no definitive evidence of malignancy (highly suspicious, dysplastic, atypia, inflammatory and normal). Endobiliary biopsy was obtained using standard endoscopic biopsy forceps. Following ERCP all patients were observed for 4 h in the recovery area prior to discharge, or admitted to hospital if further observation was deemed clinically necessary.

### Biliary brush collection and storage for MCM5 assay analysis

After removal of the biliary brush from the working channel of the endoscope the brush was advanced out of the sheath, cut and placed into a storage buffer (Varleigh Dx (UK) Ltd, London, UK), which contained one complete mini EDTA-free protease inhibitor cocktail tablet (Roche Diagnostics Ltd, Lewes, East Sussex, UK) per 10 ml of buffer. The sample was gently agitated before being rapidly frozen to −80 °C within 4 h of the procedure.

### *In vitro* diagnostic assay development and validation

**Figure 1:**
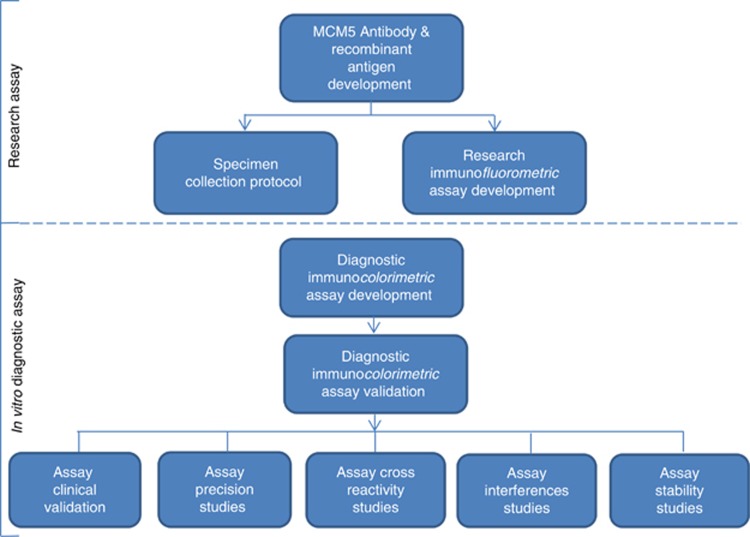
**Stages of development of the MCM5 immunocolorimetric ELISA assay.**

In conjunction with a commercial partner (Varleigh Dx (UK) Ltd) and under licence from the Cancer Research United Kingdom (CRUK) licencing group Cancer Research Technologies (CRT), a diagnostic MCM5 ELISA assay was developed in conformity with the requirements of the IVD Directive EC 98/79/EC. Validation studies were designed to test required aspects of assay performance including clinical performance, assay precision, interference, cross reactivity and stability. The pathway for translation of this assay from a research immunofluorometric method to an IVD immunocolorimetric method is shown in Figure 1.

### Antibody development

Monoclonal antibodies (MAbs) 12A7 and 4B4, directed against non-overlapping epitopes, were raised against His-tagged human MCM5 protein and were protein A-purified from hybridoma supernatants as described previously (Stoeber *et al*, 2002). For use in the ELISA assay the protein A-purified MAb 12A7 was labelled with horseradish peroxidase using conventional conjugation techniques, whereas capture antibody 4B4 was adsorbed to the surface of microtitre plate wells.

### Clinical validation studies

Ninety-seven patients with complete clinical follow-up diagnosis and adequate sample lysate volume following protein extraction were subject to the MCM5 ELISA. About 51 were performed in duplicate as per the manufacturer's instructions. The assay was controlled using (a) lysis buffer as the blank representing the cell lysate matrix and (b) recombinant MCM5 antigen-positive analyte control (concentration ∼0.60 ng ml^−1^). The assay was further controlled using a calibrator of recombinant MCM5 antigen (batch specific calibrator concentration 0.29 ng ml^−1^). Optical density (OD) measurements were recorded for all samples and controls tested. On completion of the study, patient data and the MCM5 ELISA results were compared with biliary brushing cytology results, where available, and final clinical diagnosis after follow-up.

### Assay precision studies

Precision studies were conducted utilising recombinant MCM5 antigen. A 12-day study was conducted using three reagent kit lots (12 runs with each reagent lot) and four replicates of each control sample per run.

### Assay cross reactivity and interference studies

#### Cross reactivity

In order to ensure specificity of the ELISA for the MCM5 target protein cross reactivity studies were performed against other members of the MCM family (MCM2–7). Each MCM protein was tested in duplicate over the range of concentrations 0.05–100 ng ml^−1^.

#### Interferences

During ERCP iodine-based contrast media (Omnipaque) was used to delineate the biliary tract. In our study we tested to see if Omnipaque was present in test samples at concentrations comparable to residual levels, which may be carried over into the test specimen and show interference in the assay. A worst case theoretical residual concentration of 50 μg organic iodine per ml from Omnipaque 300 (647 mg ml^−1^ iohexol/300 mg organic iodine per ml) was established and a dilution series created 50% either side of this concentration (25, 50 and 75 μg iodine per ml) in both positive and negative MCM5 solutions, measuring five replicates of each and recording the absorbance measurements at 450 nm for all samples and controls.

### Assay stability studies

In order to ensure preliminary stability of the working assay, manufactured components were assembled into kits and stability of the assay reagents were assessed by repeat measurements of a panel of samples at monthly intervals using reagents stored at 2–8 °C, using an unopened kit at each time point and also at 2 weekly intervals using a kit opened at *t*=0. The absorbance measurements at 450 nm were recorded for all samples and controls tested.

### Data analysis

Statistical package for Social Sciences for Windows, version 21.0 (SPSS Inc., Chicago, IL, USA) was used to perform all statistical analyses. Associations between various clinical and radiographic characteristics were evaluated using a 2-sample *t-*test for continuous variables and a 5% level was used to indicate significance. The sensitivity for biliary brush cytology was compared with that of the immunofluorometric MCM5 test using McNemar's test for paired proportions. Sensitivity and specificity characteristics of the MCM5 test for the detection of malignancy were presented as a receiver operating characteristics (ROC) curve. The area under the nonparametric ROC curve was used to assess the overall accuracy of the test.

## Results

### Assay precision studies

A precision profile was generated from a single assay of seven replicates of recombinant MCM5 antigen standards on each of three reagent lots. From this precision profile, CV at the cut-off concentration of 0.11 ng ml^−1^ was calculated to be 13.2%. The Total CV for Calibrator OD450 was found to be 5.7% The Total CV for Positive Control OD450 was found to be 7.0%.

### Assay cross reactivity and interference studies

#### Interference studies

The presence of iohexol and up to 75 μg iodine per ml (the components of Omnipaque) in the lysis solution had no significant effect on the test signal. The maximum effect of iohexol was seen in a 0.6 ng ml^−1^ MCM5-positive solution, when a 1.5% reduction in test signal was observed but this was still insufficient to misclassify the test result.

#### Cross reactivity

No cross reactivity was observed for proteins MCM2, 4, 6 and 7 and with percentage variation <1%. A marginal cross reactivity measured at 5.2% was observed for MCM3. Both measures were beneath the threshold of diagnostic impact.

### Assay stability studies

No change in performance was observed for the unopened kit after 7 months storage at 2–8 °C and for the opened kit after 6 weeks storage at 2–8 °C.

### Clinical validation studies: final MCM5 immunoassay performance

Biliary brush samples were acquired from 97 procedures performed on 94 patients (three patients had repeat MCM5 measurements at two different ERCPs). At recruitment to the study, 29 patients had an established diagnosis, whereas 68 were new referrals with an indeterminate stricture. Their final diagnoses are outlined in Table 1. The median age of the patients was 65 years (21–94 years). About 51 were male and 46 were female.

Of the 50 patients with malignant disease the final diagnosis was made by brush cytology (*n*=16), EUS-FNA or biopsy (*n*=3), endoscopic or endobiliary biopsy (*n*=3), cholangioscopy and biopsy (*n*=1), percutaneous pancreatic biopsy (*n*=1), percutaneous liver biopsy of metastatic disease (*n*=4), surgical resection (*n*=7), intraoperative pancreatic biopsy (*n*=2) or clinical course (*n*=13). Patients underwent a mean of 1.9 attempts at tissue acquisition (range 1–8) before malignancy was confirmed. Benign disease was confirmed by negative pathology during a median of 14 (range 0–49) months follow-up.

**Figure 2:**
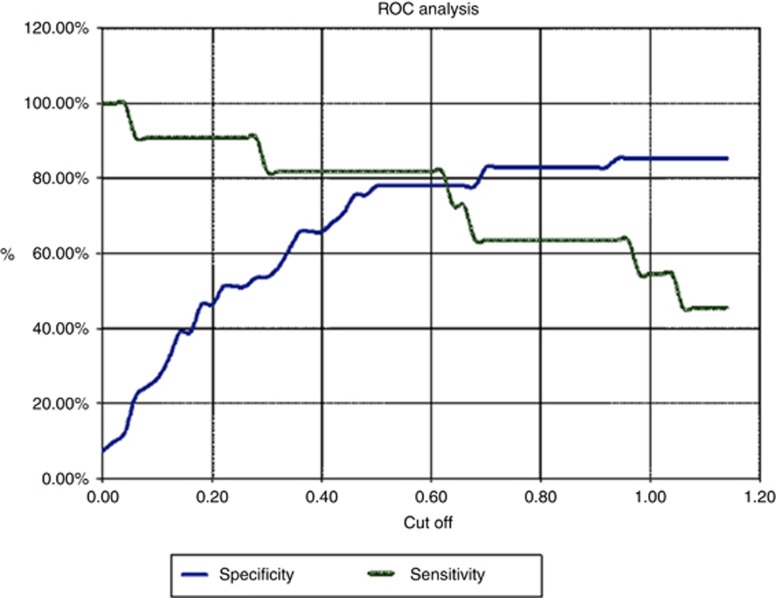
**ROC curve for patients with paired biliary brush cytology and MCM5 assay.**

The performance of the MCM5 ELISA assay as an *in vitro* diagnostic test for pancreaticobiliary malignancy in patients with indeterminate strictures is shown as a ROC curve (Figure 2). The test discriminated with high accuracy between patients with and without malignancy, as demonstrated by an area under the curve of 0.785 (95% CI 0.631–0.939), which was significantly larger than the area predicted by the null hypothesis (*P⩽*0.0001).

**Figure 3:**
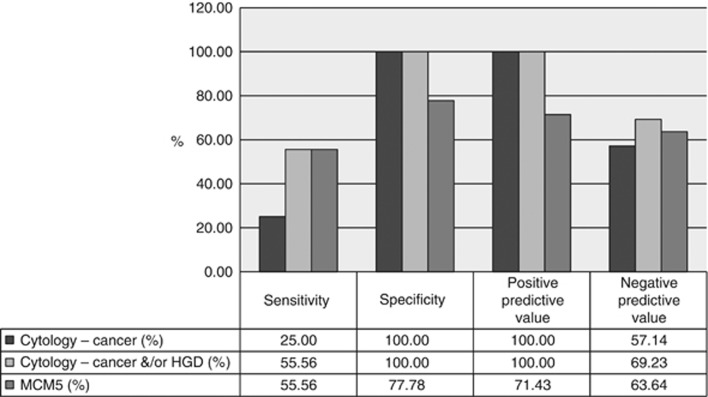
**Comparison of sensitivity, specificity, NPV and PPV in patients with paired cytology and MCM5 biliary brush assay.**

When compared directly with final diagnosis the MCM5 assay had a sensitivity and specificity of 65.38% and 77.78%, respectively. In comparison, biliary brush cytology had a sensitivity and specificity of 25.0% and 100%, respectively (Table 2, Figure 3). Differences in test performance were observed in patients with certain stricture subtypes, in particular false-positive results were seen in patients with IgG4-related disease, chronic pancreatitis and primary sclerosing cholangitis (Table 3). In the 72 patients with paired MCM5 levels and biliary brush samples, MCM5 demonstrated an improved sensitivity when compared against biliary brush cytology (55.56 *vs* 25.00% *P*=0.0002) for the detection of malignancy. No difference was seen between the sensitivity of the MCM5 assay and cytology that included cancer and high-grade dysplasia (55.56 *vs* 55.56% Figure 3).

### Cost and turnaround time analysis for MCM5 immunoassay

We estimate that a diagnostic MCM5 ELISA assay would be comparable to a non-gynaecological diagnostic cytology sample, inclusive of laboratory preparation, overheads and pathologist interpretation.

We envisage that in clinical practice the MCM5 assay would be performed on a weekly basis as a batched assay, inclusive of a LIMS interfaced report into the hospital electronic patient record. Laboratory turnaround time and cost efficiencies would be seen based on testing economies of scale. As multiple centres came online the assay could be run at least twice per week, with a turnaround time of 72 h from receipt of sample.

## Discussion

In patients who present with indeterminate biliary strictures, biliary brush cytology remains the most commonly used test to distinguish benign and malignant disease. The technique offers the clinician almost definitive diagnostic certainty when positive for malignancy (specificity 96–100%) but remains unreliable for detecting malignancy (sensitivity 9–57% De Bellis *et al*, 2002; Harewood *et al*, 2004; Moreno Luna *et al*, 2006).

We have previously shown that the upregulation of the MCM5 protein is a reliable biomarker for malignancy in tissue samples from the bladder, prostate, oesophagus and pancreaticobiliary tract (Stoeber *et al*, 2002; Williams *et al*, 2004; Ayaru *et al*, 2008). In a previous study by our group it was shown that cell cycle proteins in bile aspirates had a significantly superior diagnostic sensitivity for malignancy than routine cytology (66 *vs* 20% Ayaru *et al*, 2008). Biliary brush samples are recognised to be significantly more cellular than bile samples, so it was anticipated that samples obtained via this method would be superior to bile alone. During this validation study using biliary brush samples, a newly developed MCM5 ELISA assay was shown to be superior for detecting pancreaticobiliary malignancy over standard brush cytology while maintaining a high specificity (sensitivity of 65.38% and specificity of 77.78%).

This study confirms that measurement of the MCM5 proteins using a highly sensitivity ELISA assay is a robust method of detecting upregulated MCM5 proteins in pancreaticobiliary malignancy. Moreover, as these malignancies are associated with different sets of genetic mutations leading to uncontrolled cell proliferation, this study provides further evidence supporting the hypothesis that the convergence point of growth regulatory pathways that control cell proliferation is the initiation of genome replication in which the MCM complex plays an essential part. The expression of MCM5 in malignant biliary brush samples was equivalent to bile aspirates (Ayaru *et al*, 2008), but lower than that detected in urine and oesophageal aspirates obtained from patients with bladder and oesophageal cancer (Stoeber *et al*, 2002; Williams *et al*, 2004).

Importantly, assay performance was less accurate in certain groups, and false-positive results were seen in patients with inflammatory strictures secondary to IgG4-related disease, chronic pancreatitis and primary sclerosing cholangitis. These conditions are recognised to predispose individuals to pancreaticobiliary cancer and to be particularly difficult to distinguish from malignant disease using conventional diagnostic tests (Lazaridis and Gores, 2005; Hamer and Feuerbach, 2006). Benign strictures from bile duct stones, with or without cholangitis did express low levels of MCM5 but median levels were below the detection limit of the assay, reflecting low shedding of any reactive MCM5-positive cells. This was similar to our previous data in patients with renal calculi and oesophageal ulceration, where the test detected positive cells in urine and luminal secretions, but at a magnitude below that of patients with urothelial or oesophageal carcinoma (Stoeber *et al*, 2002; Williams *et al*, 2004). This study using a MCM5 immunofluorometric assay validates previous finding in tissue and bile aspirates in pancreaticobiliary cancer (Ayaru *et al*, 2008).

The molecular diagnosis of pancreaticobiliary malignancies has been the subject of intensive investigation (Gress, 2004), but to date very few of the tests developed have been incorporated into routine clinical practice. A recent study of FISH to detect chromosomal abnormalities in biliary brush cytology samples demonstrated results comparable to our study with a sensitivity of 59–70% and specificity of 86–100% for the diagnosis of pancreaticobiliary malignancy compared with a sensitivity of 4–20% for conventional brush cytology (Moreno Luna *et al*, 2006). Increasingly multimodal investigation of biliary strictures is advocated to enable a rapid and accurate diagnosis, so patients can commence treatment promptly (Cote *et al*, 2015). However cholangioscopy, confocal endomicrosopy (Keane *et al*, 2013), endoscopic ultrasound and in room cytopathology (De Bellis *et al*, 2002) are usually only available in specialist centres and, therefore, are only performed once standard diagnostic methods have failed to establish a definitive diagnosis. The advantage of this immunocolorimetric ELISA assay is that it is based on biliary brush sampling and is therefore technically easier to perform than the other methods described and can be done at the same time as the first diagnostic ERCP. The higher sensitivity of this test for malignancy is also likely to improve the overall diagnostic accuracy of the first ERCP procedure. It therefore has the potential to be easily incorporated into future diagnostic pathways or multimodal methods of stricture assessment.

This test also has the potential to be used in combination with other standard investigations for hepatobiliary malignancy. Our group has explored the utility of the MCM5 assay in detecting malignancy in cystic lesions of the pancreas. Early data suggest that it can detect malignancy in this setting with a sensitivity of 50% and specificity of 73% (Keane *et al*, 2016). Guidelines on the evaluation of biliary strictures increasingly advocate the use of EUS-FNA to aid diagnosis, particularly in indeterminate lesions with at least one unsuccessful attempt at tissue acquisition (Anderson *et al*, 2013). Given the promising early results using EUS-FNA in cystic lesions of the pancreas, there is the potential for the MCM5 test to be developed in the setting of EUS-FNA of pancreaticobiliary stricture assessment as well.

## Conclusions

In conclusion, we have demonstrated that a rapid, sensitive and specific immunocolorimetric ELISA for detection of MCM5 in biliary brush samples is a robust diagnostic test for pancreaticobiliary malignancy.

## Figures and Tables

**Table 1:** Final diagnosis

**Final diagnosis**	**Established diagnosis (*****n*****=29)**	**New indeterminate stricture (*****n*****=68)**	**Total (*****n*****=97)**
**Benign (*****n*****=47)**			
Primary sclerosing cholangitis	7	5	12
IgG4-related disease	6	5	11
Intraductal polyp with low-grade dysplasia	0	1	1
Benign papillary fibrosis / stone-related strictures	3	6	9
Post-surgical stricture (following liver transplant (*n*=1) or laparoscopic cholecystectomy (*n*=1))	2	0	2
Ischaemic stricture post hepatic artery thrombosis following emergency embolisation	0	1	1
Chronic pancreatitis	3	5	8
Benign cystic lesion of the pancreas	0	2	2
Caroli disease	0	1	1
**Malignant (*****n*****=50)**			
Pancreatic ductal adenocarcinoma	1	23	24
Diffuse large B-cell lymphoma of the pancreas	0	1	1
Biliary tract cancer	4	13	17
Ampullary adenocarcinoma	0	3	3
Colorectal cancer with liver metastases	1	0	1
Mediastinal non Hodgkin's lymphoma	1	0	1
Metastatic breast cancer	1	0	1
Hepatocellular carcinoma	0	1	1
Midgut neuroendocrine tumour	0	1	1

**Table 2:** Comparison of sensitivity, specificity, NPV and PPV for biliary brush cytology, MCM5 assay, a combination of cytology and MCM5 assay and the MCM5 assay excluding patients with IgG4-related disease

**Patient group**	**Group size (*****N***)	**Sensitivity (%)**	**Specificity (%)**	**Positive predictive value (%)**	**Negative predictive value (%)**
Biliary brush cytology	72	25.00 (12.12–42.20)	100.00 (90.26–100.00)	100.00 (66.37–100.00)	57.14 (44.05–69.54)
MCM5 assay	97	65.38 (50.91–78.03)	77.78 (62.91–88.80)	77.27 (62.16–88.53)	66.04 (51.73–78.48)
Cytology+MCM5 assay	72	64.71 (46.49–80.25)	78.95 (62.68–90.45)	73.33 (54.11–87.72)	71.43 (55.42–84.28)
MCM5 assay (excluding patients with IgG4-RD)	86	62.00 (47.17–75.35)	80.56 (63.98–91.81)	81.58 (65.67–92.26)	60.42 (45.27–74.23)

Abbreviations: IgG4-RD=IgG4-related disease; NPV=negative predictive value; PPV=positive predictive value.

**Table 3:** MCM5 test performance in individual subgroups

**Patient group**	**Group size (*****N***)	**Median signal**	**Range**	**False positive**	**False negative**
Benign	47	0.205	(0.003–11.868)	12/47	0/47
IgG4-related disease	11	0.169	(0.066–8.916)	5/11	0/11
Chronic pancreatitis	9	0.202	(0.019–1.868)	3/9	0/8
Primary sclerosing cholangitis	12	0.32	(0.003–1.67)	2/12	0/12
Benign papillary fibrosis / stone disease / post-surgical strictures	11	0.0116	(0.072–8.352)	1/11	0/11
Polypoid intraductal biliary lesion	1	0.1018		1/1	
Malignant	50	0.892	(0.007–24.795)	0/50	19/50
Pancreatic ductal adenocarcinoma	24	0.6915	(0.024–4.792)	0/24	9/24
Biliary tract cancer	17	1.111	(0.007–24.795)	0/17	6/17
Ampullary adenocarcinoma	3	0.393	(0.213–2.622)	0/3	2/3
Non-HPB cancer	5	2.08	(0.121–12.392)	0/5	1/5
Diffuse large B-cell lymphoma of the pancreas	1	0.0927			1/1

Abbreviation: HPB=hepatopancreaticobiliary.
